# Supplementary material for: Identification and functional analysis of non-coding regulatory small RNA FenSr3 in Bacillus amyloliquefaciens LPB-18
Source: PeerJ. 2023 May 15;11:e15236. doi: 10.7717/peerj.15236 (PMC10194069; doi:10.7717/peerj.15236)
Supplement: Supplemental Information 4 [file peerj-11-15236-s004.zip › KO/CK-vs-T1_map/map04270.html]

KEGG PATHWAY: Vascular smooth muscle contraction - Reference pathway


|  |  |
| --- | --- |
| **Vascular smooth muscle contraction - Reference pathway** |  |

[
Pathway menu
| Organism menu
| Pathway entry
| Show description
| User data mapping
]

The vascular smooth muscle cell (VSMC) is a highly specialized cell whose principal function is contraction. On contraction, VSMCs shorten, thereby decreasing the diameter of a blood vessel to regulate the blood flow and pressure.
The principal mechanisms that regulate the contractile state of VSMCs are changes in cytosolic Ca2+ concentration ([Ca2+]c). In response to vasoconstrictor stimuli, Ca2+ is mobilized from intracellular stores and/or the extracellular space to increase [Ca2+]c in VSMCs. The increase in [Ca2+]c, in turn, activates the Ca2+-CaM-MLCK pathway and stimulates MLC20 phosphorylation, leading to myosin-actin interactions and, hence, the development of contractile force. The sensitivity of contractile myofilaments or MLC20 phosphorylation to Ca2+ can be secondarily modulated by other signaling pathways. During receptor stimulation, the contractile force is greatly enhanced by the inhibition of myosin phosphatase. Rho/Rho kinase, PKC, and arachidonic acid have been proposed to play a pivotal role in this enhancement.
The signaling events that mediate relaxation include the removal of a contractile agonist (passive relaxation) and activation of cyclic nucleotide-dependent signaling pathways in the continued presence of a contractile agonist (active relaxation). Active relaxation occurs through the inhibition of both Ca2+ mobilization and myofilament Ca2+ sensitivity in VSMCs.

|  |  |  |
| --- | --- | --- |
| Reference pathway | 184% 150% 122% 100% 82% 67% 55% | 图片下载 |
